# Supplementary material for: Self-rechargeable cardiac pacemaker system with triboelectric nanogenerators
Source: Nat Commun. 2021 Jul 16;12:4374. doi: 10.1038/s41467-021-24417-w (PMC8285394; doi:10.1038/s41467-021-24417-w)
Supplement: Supplementary file 1 — Supplementary Information [file 41467_2021_24417_MOESM1_ESM.pdf]

SUPPLEMENTARY MATERIALS

Self-rechargeable cardiac pacemaker system with triboelectric nanogenerators

Hanjun Ryu[1†], Hyun-moon Park[2†], Moo-Kang Kim[3], Bosung Kim[1], Hyoun Seok Myoung[2], Tae Yun Kim[1], Hong-Joon Yoon[1], Sung Soo Kwak[1], Jihye Kim[1], Tae Ho Hwang[4], Eue-Keun Choi[3] and Sang-Woo Kim[1,5*]

[1]School of Advanced Materials Science and Engineering, Sungkyunkwan University (SKKU), Suwon, 16419, Republic of Korea.

[2]Research and Development Center, Energy-Mining LTD., Seoul, 06555, Republic of Korea.

[3]Department of Internal Medicine, Seoul National University Hospital, Seoul, 03080, Republic of Korea.

[4]SoC Platform Research Center, Korea Electronics Technology Institute (KETI), Seongnam, 13509, Republic of Korea.

[5]SKKU Advanced Institute of Nanotechnology (SAINT) and Samsung Advanced Institute for Health Sciences & Technology (SAIHST), Sungkyunkwan University (SKKU), Suwon 16419, Republic of Korea.

[†]These authors contributed equally to this work.

[*]E-mail: kimsw1@skku.edu (S.-W. Kim)

Note 1. Analysis of the amine-functionalized poly(vinyl alcohol).

In order to confirm amine functionalization, chemical modification of PVA was monitored by Fourier transform infrared spectroscopy (FT-IR). Supplementary Fig. 1a presents the FT-IR spectrum of PVA-NH_2. A large peak at 3300 cm^{-1} was the stretching of O-H, a peak at 1920 cm^{-1} was the stretching vibration of C-H from the alkyl group, and a peak at 1590 cm^{-1} indicated primary amine function.

Kelvin probe force microscopy (KPFM) was used to measure the surface potential of PVA-NH_2 (see Supplementary Fig. 1b). The surface potential of a material is one of the important factors that determine its triboelectric properties. The surface potential of pristine PVA was 247 mV[22], and that of amine-functionalized PVA was 505 mV. Thus, the increased surface potential of PVA-NH_2 had a more positive triboelectric property than did pristine PVA.

In order to verify the PVA-NH_2 mechanical coating property, a steel wool abrasion test was performed for 500 cycles with #0000 steel wool under a load weight of 250 g and a scratch velocity of 5 mm/s (see Supplementary Fig. 1c). After 500 cycles, the PVA-NH_2 did not have significant detachment but only shallow scratches. A pencil hardness test with a load weight of 350 g and a velocity of 1.75 mm/s was also performed to verify the hardness of the PVA-NH_2 (see Supplementary Fig. 1d). A pencil of 9H hardness made a slight indentation on the PVA-NH_2. Therefore, the PVA-NH_2 film had strong adhesion and mechanical durability.

Note 2. The working mechanism of the I-TENG.

To identify the working mechanism of the I-TENG, a detailed cyclic process was used, as shown in Supplementary Fig. 2. It depended on the movement of the freestanding unit without the initial contact electrification process. The PFA layer has negative triboelectric charges on its surface, the PVA-NH_2 has positive triboelectric charges, the Cu electrode has positive charges to neutralize the negative triboelectric charges on the PFA, and the top and bottom Au electrodes have negative charges to neutralize the positive triboelectric charges on the PVA-NH_2. During the initial stage, when the body moves vertically upwards, the I-TENG moves up with the body. At this time, all individual parts have the same vertical velocity, so the freestanding unit is not separated from the bottom PVA-NH_2 (see Supplementary Fig. 2a). When the vertical body motion slows, the freestanding unit detaches vertically from the bottom PVA-NH_2 layer due to inertia, while the package is slowed by human motion (see Supplementary Fig. 2b). To reach equilibrium, electrons flow from the Cu mass to the bottom Au electrode through an external circuit. A specially designed structure limits the maximum displacement of the freestanding unit as it collides with the top PVA-NH_2 layer. As the freestanding unit approaches the top PVA-NH_2 layer, negative charges on the top Au electrode are neutralized, and electrons flow from the top Au electrode to the Cu through the external circuit until electrical equilibrium is reached (see Supplementary Fig. 2c, 2d). Since the freestanding unit loses vertical upward inertia and the body moves vertically downwards, the freestanding unit is separated from the top PVA-NH_2 layer by gravity (see Supplementary Fig. 2e). To reach equilibrium again, electrons flow from the Cu to the top Au electrode through the external circuit in all I-TENGs. After the freestanding unit contacts the bottom PVA-NH_2 layer, negative charges on the bottom Au electrode are neutralized, and electrons flow into the Cu via the external circuit until equilibrium is reached (see Supplementary Fig. 2f, 2a).

Note 3. Mass, gap, and surface design of the I-TENG.

Electrostatic attraction force between the positive and negative triboelectric layers should be weaker than the applied inertial force in order to separate the two materials. The electrostatic attraction force ($F_{Electrostatic}$) and applied inertial force ($F_{Inertia}$) of the device are given as

$$F_{Inertia} > F_{Electrostatic} \ (1)$$

$$F_{Electrostatic} = \frac{Q^2}{2\varepsilon_0 \varepsilon_{r,air} A} = \frac{(42\times10^{-9})^2}{2\times8.85\times10^{-12}\times4.9\times10^{-4}} = 0.205\ N \ (2)$$

$$F_{Inertia} = ma = 3.318 \times 10^{-3} \times 8.22 \times 9.8 = 0.256\ N \ (3)$$

where Q is the surface triboelectric charge, ε_0 is vacuum permittivity, $\varepsilon_{r,air}$ is relative air permittivity, A is the surface area of the triboelectric layer, m is the weight of the Cu mass, and a is acceleration. When the thickness of the Cu is above 0.7 mm and acceleration is 8.22 g, $F_{Inertia}$ is larger than $F_{Electrostatic}$. Therefore, the Cu mass should be thicker than 0.7 mm (see Supplementary Fig. 4a).

In order to identify the optimal gap distance between the positive and negative triboelectric layers, we assessed charge transfer efficiency depending on the gap distance, expressed as

$$\text{Charge transfer efficiency} = \frac{\Delta\sigma}{\sigma} = \frac{z}{d_{PFA}\varepsilon_0/\varepsilon_{PFA} + d_{PVA-NH_2}\varepsilon_0/\varepsilon_{PVA-NH_2} + z} \times 100(\%) \ (4)$$

where σ is the triboelectric surface charge, d is the thickness, ε is relative permittivity, ε_0 is vacuum permittivity, and z is the gap distance between the top and bottom triboelectric materials. As shown in Supplementary Fig. 4b, charge transfer efficiency was related to gap distance, and 1 mm of gap distance secures a 99% charge transfer efficiency. Thus, a 1 mm gap distance between the positive and negative triboelectric layers is sufficient.

The output voltage of the I-TENGs is strongly affected by surface area, so increasing the surface area by surface treatment is an efficient strategy for increasing output voltage. Output voltage at a load resistance of $V_{load}(t)$ is expressed as

$$V_{load}(t) = RI_{load}(t) = R^2 A \frac{d\sigma(z,t)}{dt} \ (5)$$

where R is the load resistance, $I_{load}(t)$ is output current at a load resistance, A is the surface area, and $\frac{d\sigma(z,t)}{dt}$ is a gap- and time-dependent surface charge density. COMSOL Multiphysics

simulation results showed that micro/nano surface treatment of the PFA increased the total surface area, which resulted in a higher output voltage than that of untreated PFA (see Supplementary Fig. 4c and 4d).

Note 4. Calculation of the internal energy conversion efficiency.

In order to estimate the kinetic behavior of the five-stacked I-TENG, the velocity of the I-TENG was calculated using the measured acceleration of the I-TENG (see Supplementary Fig. 10). The input kinetic energy of the I-TENG is derived as

$$\text{Input kinetic energy} = \left[\frac{1}{2}mv^2\right]_{v_{\text{initial}}}^{v_{max}} = \frac{1}{2} \times 28.5\ (g) \times 0.34\ (m^2s^{-2}) = 4.845\ mJ \quad (6)$$

Output electric energy of the I-TENGs is expressed as

$$\text{Output electric energy} = W_{RMS} \times (t_1 - t_0) = 38.1\ (\mu W) \times 0.3\ (s) = 11.43\ \mu J \quad (7)$$

Therefore, the internal energy conversion efficiency of the I-TENG can be defined as the ratio between the output electric energy and the input kinetic energy of the I-TENG, which is expressed as

$$\text{Internal energy conversion efficiency} = \frac{\text{Output electric energy}}{\text{Input kinetic energy}} = \frac{11.43}{4845} \times 100 = 0.235(\%) \quad (8)$$

Therefore, the internal energy conversion efficiency of the I-TENG is 0.235%.

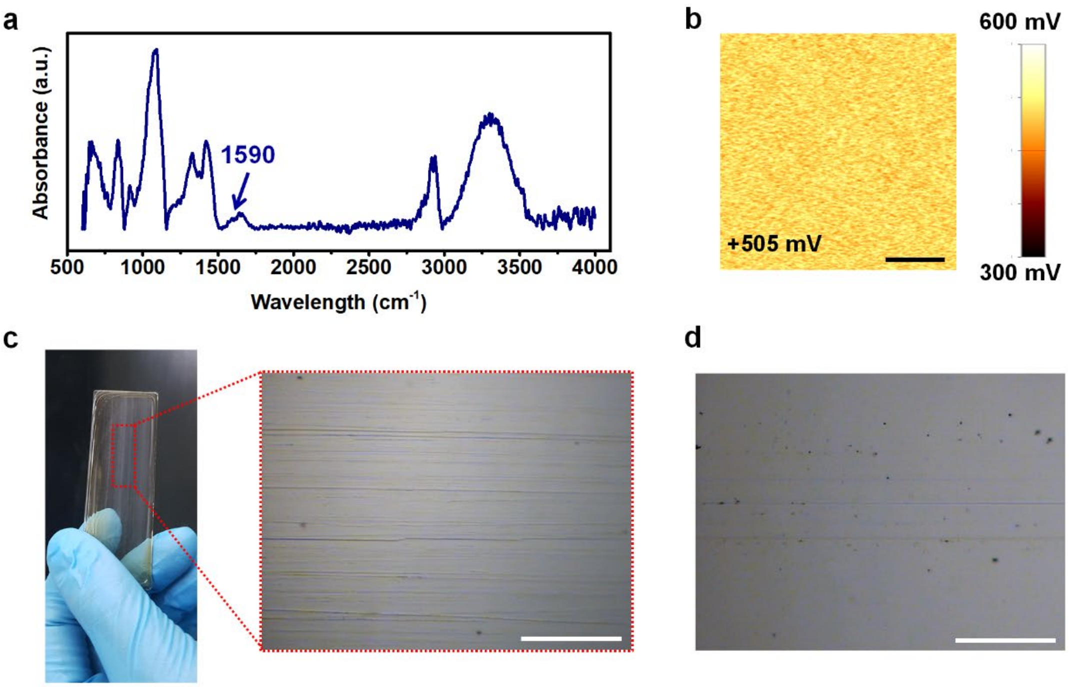

Fig. 1 Property of the amine-functionalized poly(vinyl alcohol). (**a**) FT-IR result of the PVA-NH_2 thin film. (**b**) KPFM image of the PVA-NH_2 thin film. scale bar, 5 μm. (**c**) A photographic image of the steel wool abrasion-tested PVA-NH_2. An inset optical microscopy image shows the surface of the PVA-NH_2 thin film. scale bar, 5 mm. (**d**) An optical microscopy image after the 9H pencil hardness test of the PVA-NH_2 thin film. scale bar, 5 mm. Three repeated experiments were performed.

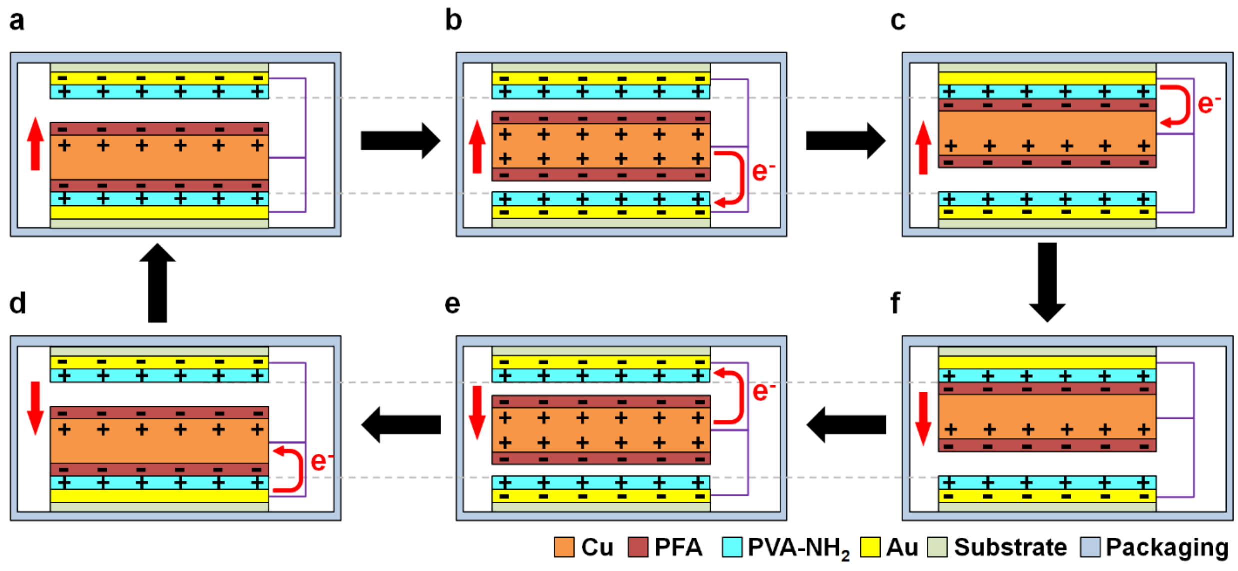

Fig. 2 The working mechanism of the I-TENG driven by biomechanical energy. (**a**) The initial state, in which all parts move upward. (**b**) The intermediate state, in which the freestanding unit moves upward. (**c**) The final state, in which the freestanding unit contacts the PVA-NH_2 layer. (**d**) The second initial state, in which the freestanding unit begins to move downward due to gravity. (**e**) The intermediate state, in which the freestanding unit moves downward. (**f**) The second final state, in which the freestanding unit contacts the PVA-NH_2 layer.

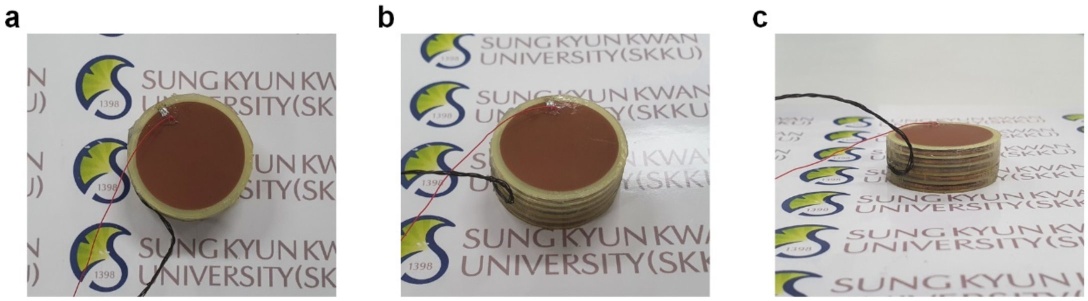

Fig. 3 Photo images of the five-stacked I-TENGs from (a) top, (b) tilted, and (c) side views.

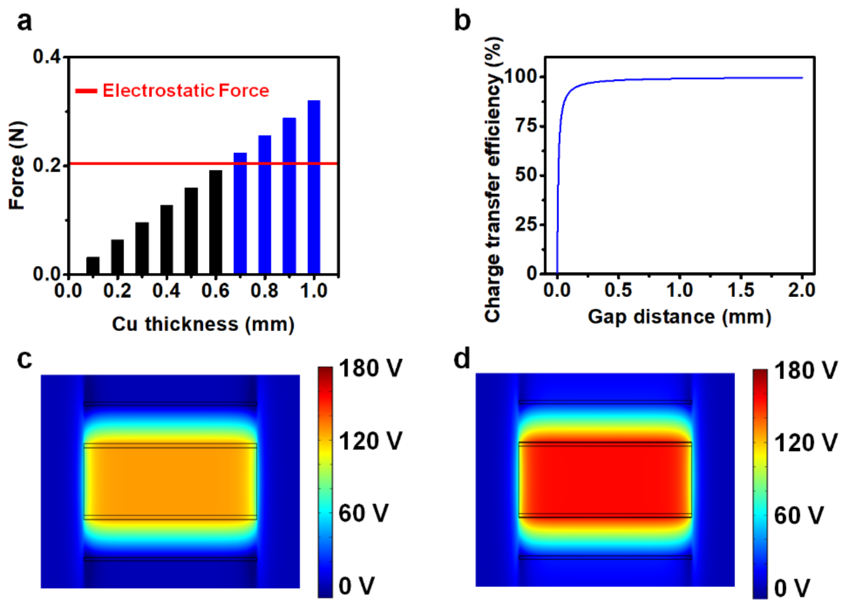

Fig. 4 Mass, gap, and surface design of the I-TENG. (**a**) The kinetic force of the freestanding unit depends on the thickness of Cu. (**b**) The charge transfer efficiency depends on the gap distance of the I-TENG. Voltage potential distribution of the I-TENG using (**c**) untreated PFA and PVA-NH_2 and (**d**) treated PFA and PVA-NH_2 as triboelectric materials.

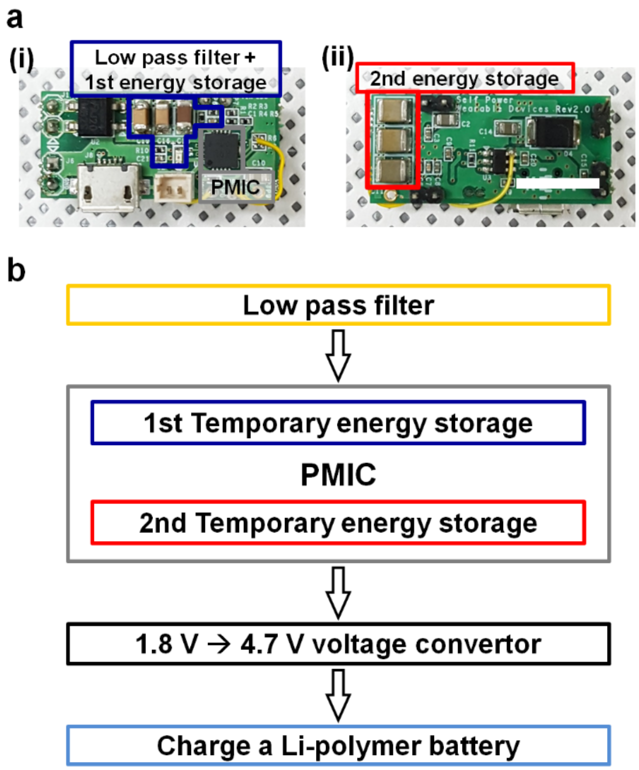

Fig. 5 The power management system. (**a**) Photographs of the (i) front and (ii) back of the power management system. scale bar, 1 cm. (**b**) System-level block diagram of the power management system for the I-TENG.

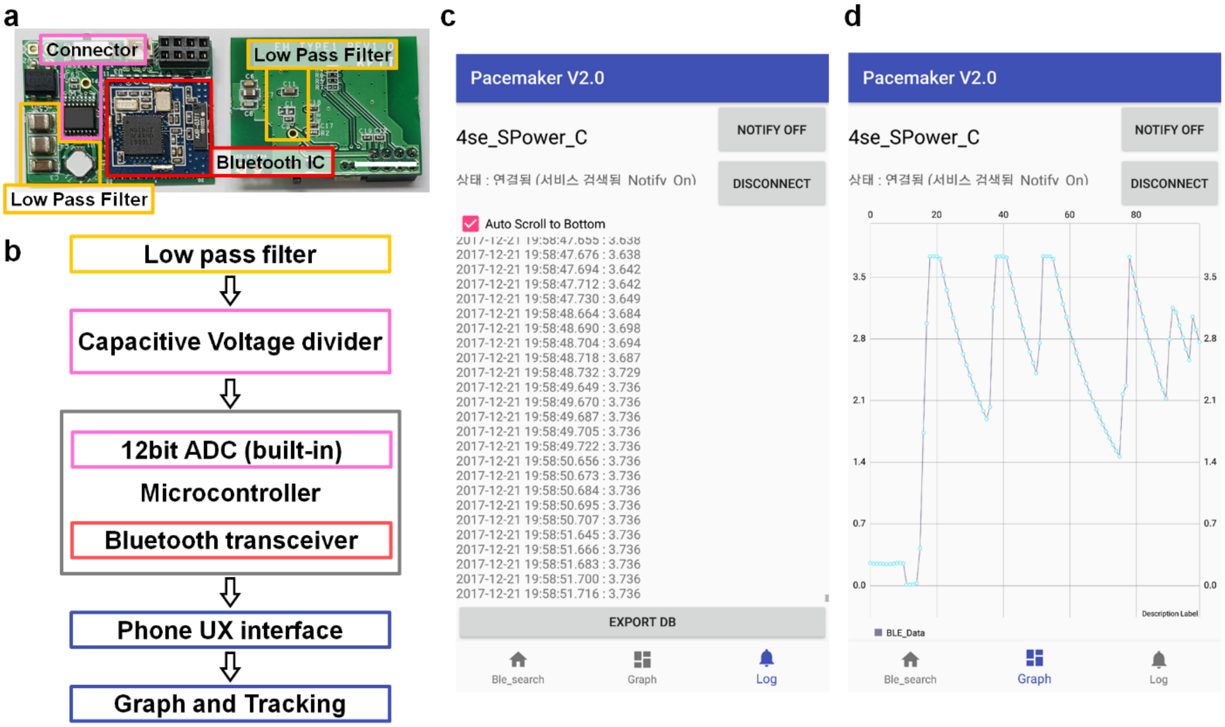

Fig. 6 The wireless monitoring system. (**a**) A photograph of the wireless monitoring system. scale bar, 1cm. (**b**) System-level block diagram of the wireless monitoring system. Screenshot of the (**c**) log and (**d**) graph page showing real-time output voltage information of the I-TENG displayed in a mobile application.

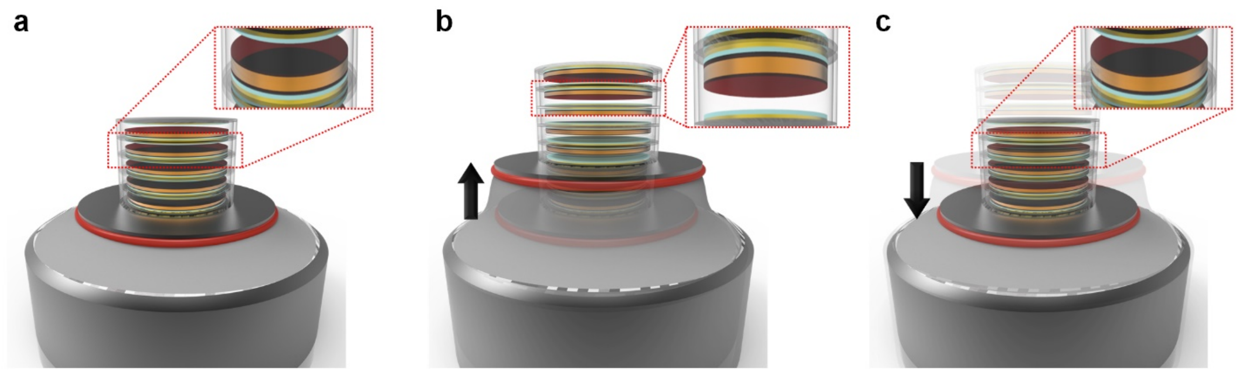

Fig. 7 Schematic process of applying kinetic energy. (**a**) The initial condition of the I-TENG on an electrodynamic transducer. (**b**) Application of upward energy to the I-TENG with the electrodynamic transducer. (**c**) The final state of the I-TENG on the electrodynamic transducer.

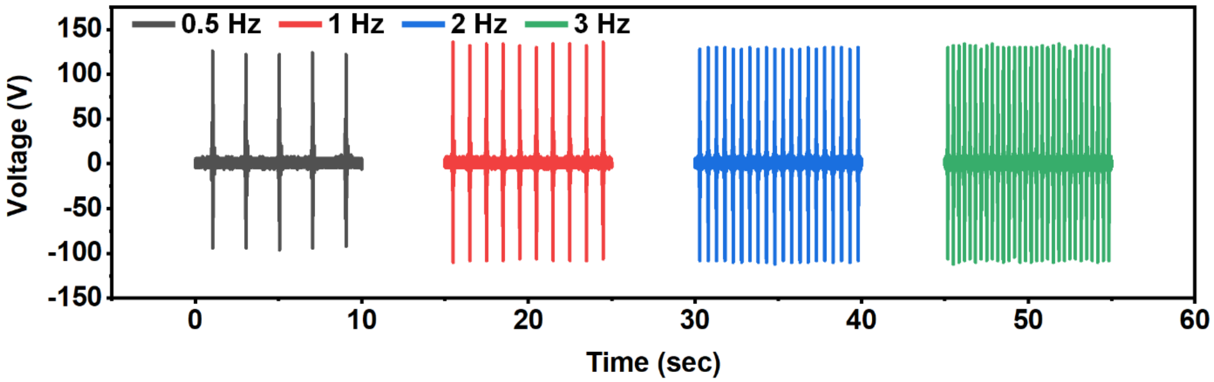

Fig. 8 Output voltage performance of the I-TENG as a function of a frequency range from 0.5 to 3 Hz.

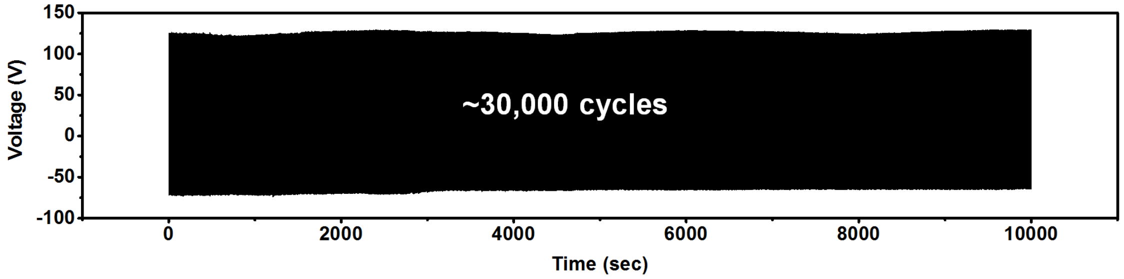

Fig. 9 Durability of the five-stacked I-TENG over 30,000 cycles.

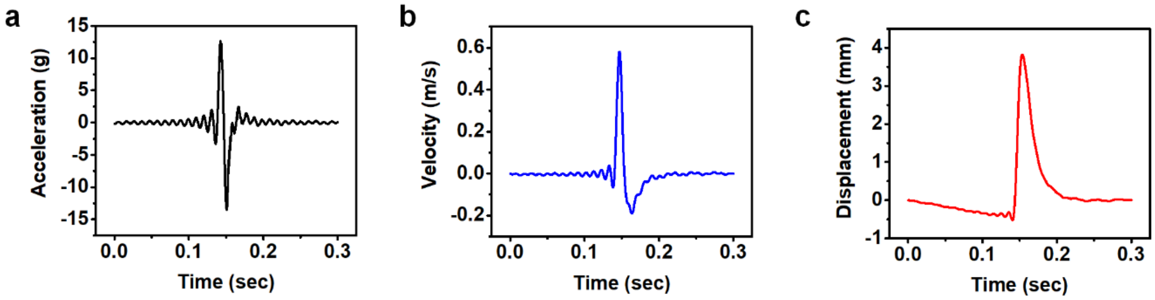

Fig. 10 Vertical input kinetic information of the I-TENG. (**a**) Vertical acceleration of the I-TENG. (**b**) Vertical velocity of the I-TENG. (**c**) Vertical displacement of the I-TENG.

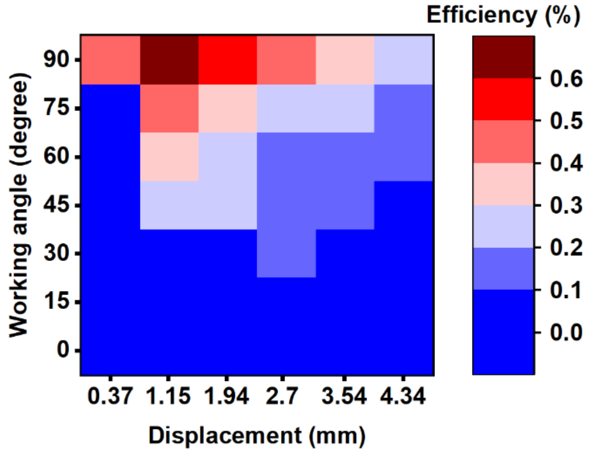

Fig. 11 The energy conversion efficiency of the I-TENG depending on different displacement and working angle conditions.

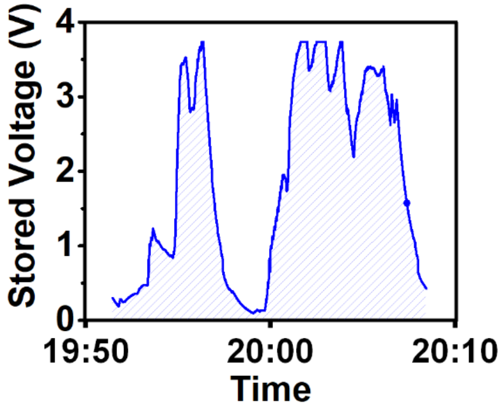

Fig. 12 Real-time energy harvesting performance of the I-TENGs while walking, running, climbing stairs, and resting.

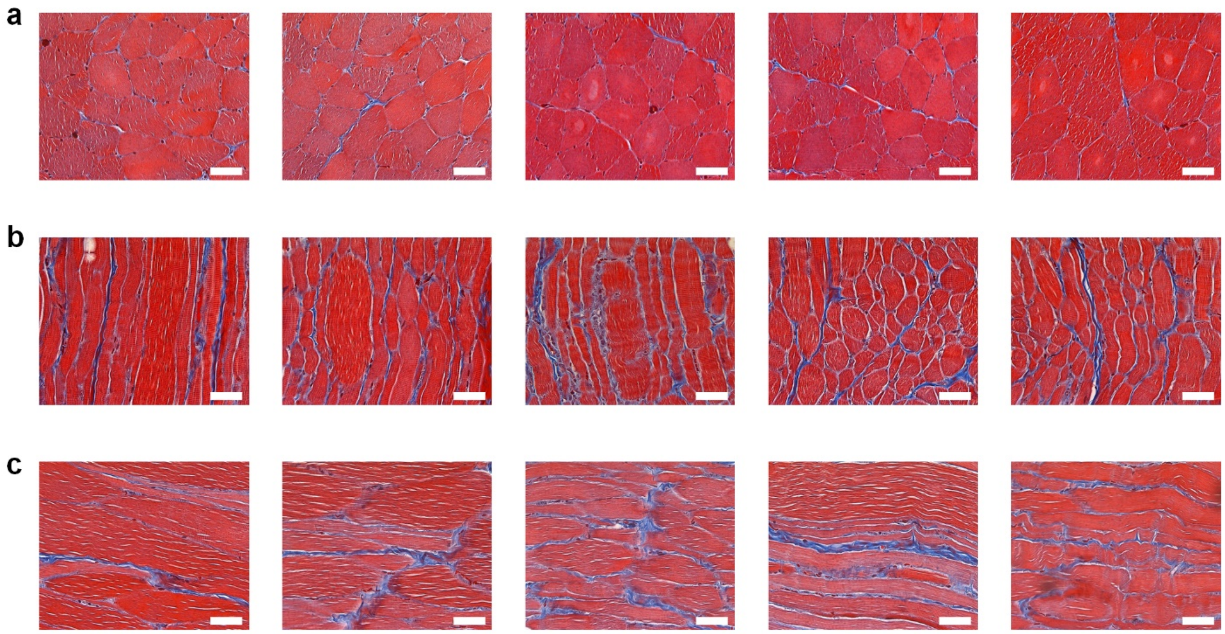

Fig. 13 Masson's trichrome stain results. Masson's trichrome stain results: (a) no inflammatory cells and scanty amount of perivascular fibrosis observed in normal muscle; (b) prominent fibrosis and a mild inflammatory response around the encapsulated device; and (c) similar fibrosis and inflammatory response around a commercial medical device. Red, white, and blue represent normal muscle, fat, and infection, respectively. scale bar, 50 μm. The Masson's trichrome stain results randomly selected 5 groups of three different conditions.

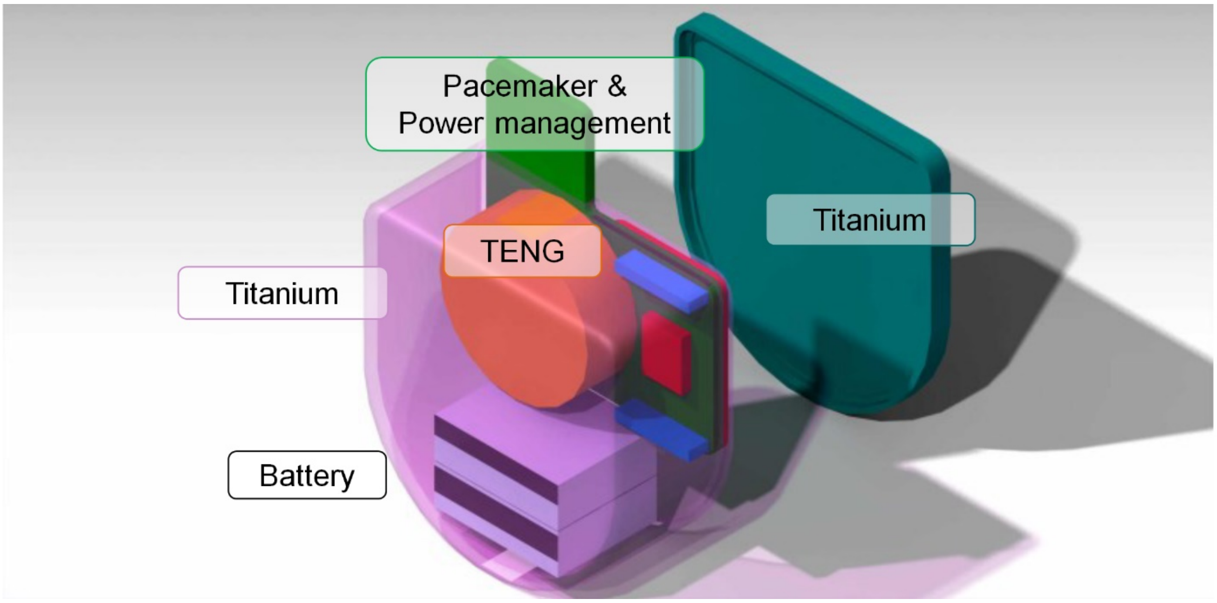

Fig. 14 Schematic structural image of the self-rechargeable cardiac pacemaker.

a

Mode	VOO
VP (V)	5
VPW (ms)	1
VS (mV)	1
LRI (bpm)	150
AVI (ms)	150
PVARP (ms)	400
VRP (ms)	250
Sampling (Hz)	200

b

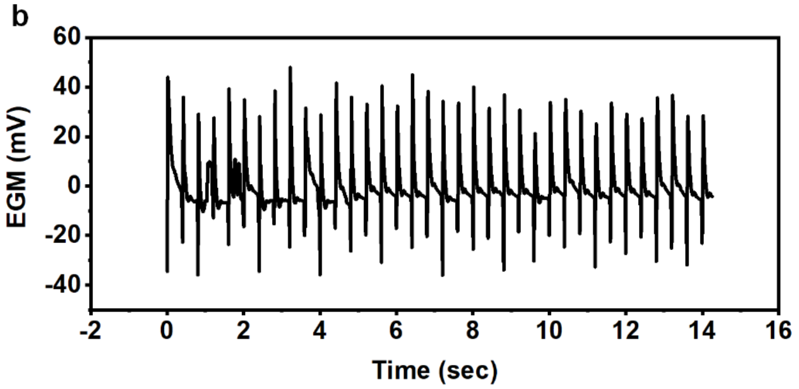

Fig. 15 VOO mode operation of the self-rechargeable pacemaker. (**a**) Parameters of the self-powered cardiac pacemaker. (**b**) Ventricular EGM signals during ventricle pacing at 150 bpm.

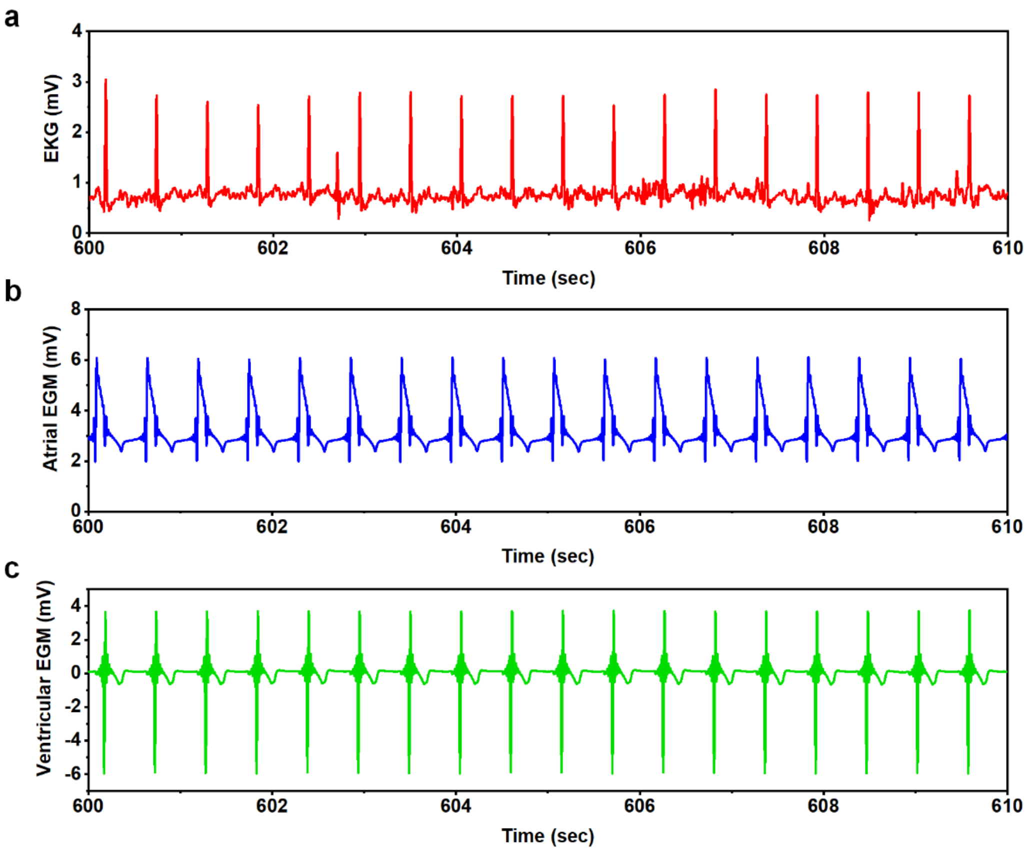

Fig. 16 Electrogram signals in the normal condition. (**a**) Electrokardiogram (EKG), (**b**) atrial EGM, and (**c**) ventricle EGM signals.

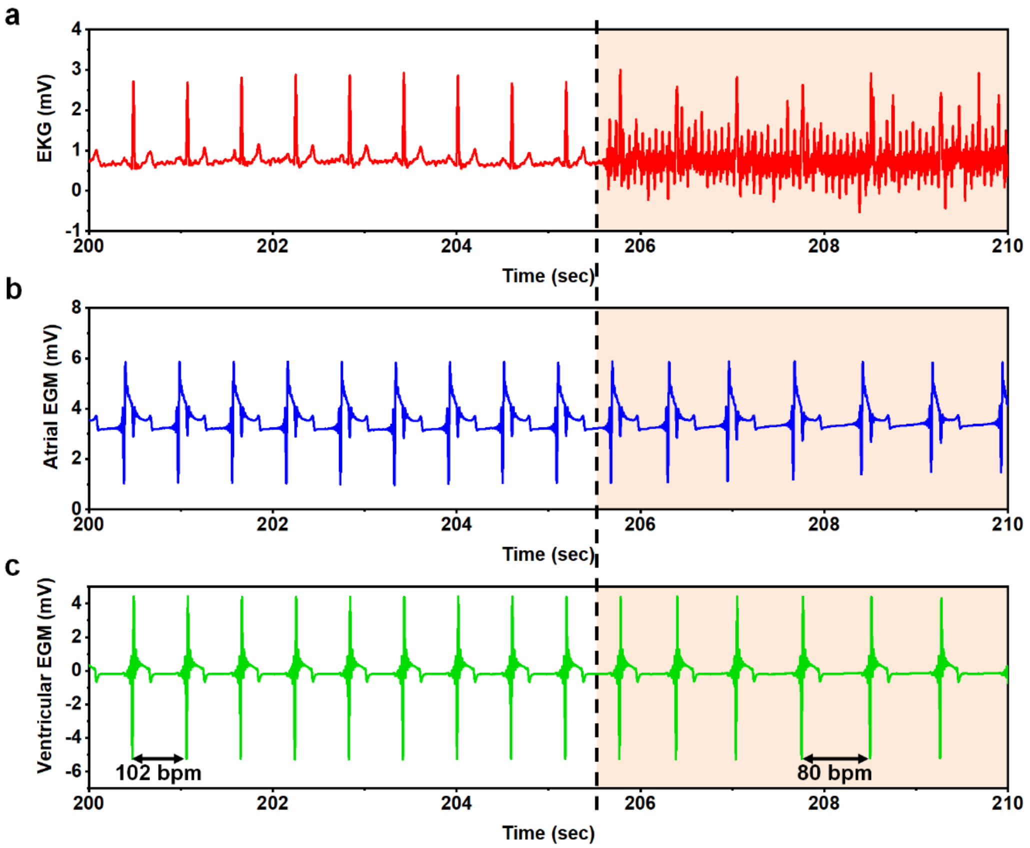

Fig. 17 Electrogram signals before and after adenosine injection. (**a**) EKG, (**b**) atrial EGM, and (**c**) ventricle EGM signals.

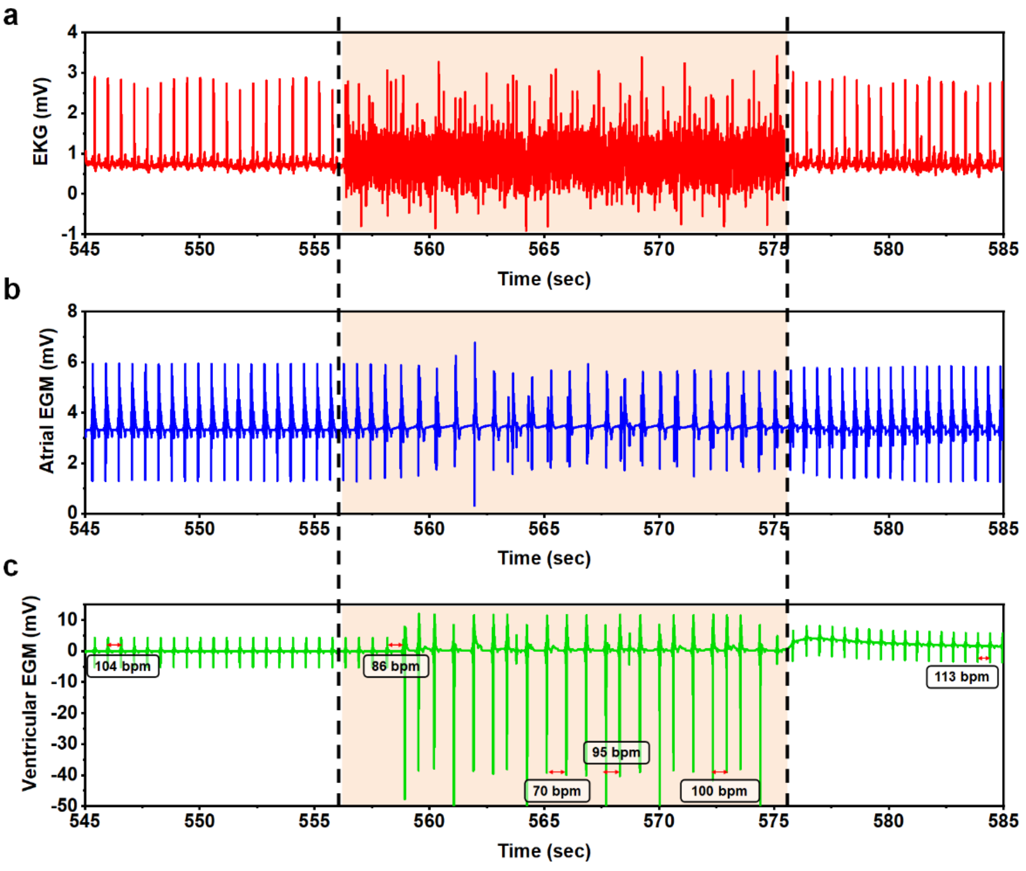

Fig. 18 Electrogram signals with pacemaker during temporary bradycardia. (**a**) EKG, (**b**) atrial EGM, and (**c**) ventricle EGM signals.

Subject		Objects	Objects(%)	Area(%)
Reference (x12.5)	base	294	24.277456	89.752327
	fibrosis	917	75.722542	10.247671
Reference (x400)-1	base	372	17.953669	91.684433
	fibrosis	1700	82.046333	8.3155689
Reference (x400)-2	base	103	17.820068	97.480957
	fibrosis	475	82.179932	2.5190461
Reference (x400)-3	base	434	29.265003	94.023598
	fibrosis	1049	70.734993	5.9764004
Reference (x400)-4	base	520	37.708485	97.330269
	fibrosis	859	62.291515	2.6697299
Reference (x400)-5	base	823	77.714828	99.09906
	fibrosis	236	22.285175	0.90094179
Encapsulated device (x12.5)	base	284	10.179212	81.966362
	fibrosis	2506	89.820786	18.03364
Encapsulated device (x400)-1	base	1246	42.337749	70.49247
	fibrosis	1697	57.662251	29.507532
Encapsulated device (x400)-2	base	459	29.291639	78.428154
	fibrosis	1108	70.708359	21.571846
Encapsulated device (x400)-3	base	621	56.148281	74.556267
	fibrosis	485	43.851719	25.443731
Encapsulated device (x400)-4	base	738	29.914877	77.775246
	fibrosis	1729	70.085121	22.224751
Encapsulated device (x400)-5	base	691	39.942196	89.024048
	fibrosis	1039	60.057804	10.975951
Commercial medical device (x12.5)	base	161	11.641359	80.368134
	fibrosis	1222	88.358643	19.63187
Commercial medical device (x400)-1	base	545	40.580788	87.909172
	fibrosis	798	59.419212	12.090829
Commercial medical device (x400)-2	base	603	33.077347	76.231331
	fibrosis	1220	66.922653	23.768671
Commercial medical device (x400)-3	base	198	22.34763	76.65596
	fibrosis	688	77.652367	23.344038
Commercial medical device (x400)-4	base	428	23.790995	75.031876
	fibrosis	1371	76.209007	24.968126
Commercial medical device (x400)-5	base	309	25.203915	79.470139
	fibrosis	917	74.796082	20.529861

Table 1. The Masson's trichrome stain results.
