# Supplementary material for: Self-rechargeable cardiac pacemaker system with triboelectric nanogenerators
Source: Nat Commun. 2021 Jul 16;12:4374. doi: 10.1038/s41467-021-24417-w (PMC8285394; doi:10.1038/s41467-021-24417-w)
Supplement: Supplementary file 3 — Reporting Summary [file 41467_2021_24417_MOESM3_ESM.pdf]

nature research

Corresponding author(s): Sang-Woo Kim

Last updated by author(s): May 24, 2021

Reporting Summary

Nature Research wishes to improve the reproducibility of the work that we publish. This form provides structure for consistency and transparency in reporting. For further information on Nature Research policies, see our Editorial Policies and the Editorial Policy Checklist.

Statistics

For all statistical analyses, confirm that the following items are present in the figure legend, table legend, main text, or Methods section.

n/a	Confirmed	
☐	☒	The exact sample size (*n*) for each experimental group/condition, given as a discrete number and unit of measurement
☒	☐	A statement on whether measurements were taken from distinct samples or whether the same sample was measured repeatedly
☐	☒	The statistical test(s) used AND whether they are one- or two-sided<br>*Only common tests should be described solely by name; describe more complex techniques in the Methods section.*
☒	☐	A description of all covariates tested
☒	☐	A description of any assumptions or corrections, such as tests of normality and adjustment for multiple comparisons
☐	☒	A full description of the statistical parameters including central tendency (e.g. means) or other basic estimates (e.g. regression coefficient) AND variation (e.g. standard deviation) or associated estimates of uncertainty (e.g. confidence intervals)
☐	☒	For null hypothesis testing, the test statistic (e.g. *F*, *t*, *r*) with confidence intervals, effect sizes, degrees of freedom and *P* value noted<br>*Give P values as exact values whenever suitable.*
☒	☐	For Bayesian analysis, information on the choice of priors and Markov chain Monte Carlo settings
☒	☐	For hierarchical and complex designs, identification of the appropriate level for tests and full reporting of outcomes
☒	☐	Estimates of effect sizes (e.g. Cohen's *d*, Pearson's *r*), indicating how they were calculated

Our web collection on statistics for biologists contains articles on many of the points above.

Software and code

Policy information about availability of computer code

Data collection	Tektronix oscilloscope, ADInstrument Powerlab, Custom smartphone application
Data analysis	Origin 2018, Graphpad ver 6.0, ImageJ, LabChart 8

For manuscripts utilizing custom algorithms or software that are central to the research but not yet described in published literature, software must be made available to editors and reviewers. We strongly encourage code deposition in a community repository (e.g. GitHub). See the Nature Research guidelines for submitting code & software for further information.

Data

Policy information about availability of data

All manuscripts must include a data availability statement. This statement should provide the following information, where applicable:

- Accession codes, unique identifiers, or web links for publicly available datasets
- A list of figures that have associated raw data
- A description of any restrictions on data availability

The data that support the findings of this study are available from the corresponding author upon reasonable request.

Field-specific reporting

Life sciences study design

All studies must disclose on these points even when the disclosure is negative.

Sample size	The sample (adult mongrel dog) size was chosen to be 3 to validate the functionality of the implantable triboelectric nanogenerators and self-rechargeable cardiac pacemaker system, and it was sufficient as the results were reproducible and demonstrated device capabilities. One sample was used to evaluate the I-TENG, and another sample was used to evaluate the capacitor charging behavior. The last sample was used to evaluate the capacitor charging behavior and self-rechargeable cardiac pacemaker property.<br>The Masson's trichrome stain results randomly selected 5 groups of three different conditions.
Data exclusions	No data were excluded.
Replication	All attempts at replication were successful. Every experiment, the I-TENG generated power successfully.
Randomization	Randomization was not relevant to this study as the primary aim was to demonstrate the functionality of the devices. Every device operates properly and output performances are similar. This experiments do not affect by biological condition of animal.
Blinding	Blinding was not relevant to this study. The device is completely isolated and a blinding process would nor affect the results.

Reporting for specific materials, systems and methods

We require information from authors about some types of materials, experimental systems and methods used in many studies. Here, indicate whether each material, system or method listed is relevant to your study. If you are not sure if a list item applies to your research, read the appropriate section before selecting a response.

Materials & experimental systems

n/a	Involved in the study	
☒	☐	Antibodies
☒	☐	Eukaryotic cell lines
☒	☐	Palaeontology and archaeology
☐	☒	Animals and other organisms
☐	☒	Human research participants
☒	☐	Clinical data
☒	☐	Dual use research of concern

Methods

n/a	Involved in the study	
☒	☐	ChIP-seq
☒	☐	Flow cytometry
☒	☐	MRI-based neuroimaging

Animals and other organisms

Policy information about studies involving animals; ARRIVE guidelines recommended for reporting animal research

Laboratory animals	adult mongrel, male, 25 to 30 kg, 6 to 12 month old.
Wild animals	This study did not involve wild animals.
Field-collected samples	This study did not involve field-collected samples.
Ethics oversight	The protocol for this study was approved by the Institutional Animal Care and Use Committee of the Seoul National University Hospital, and the animals were maintained in the facility accredited by AALAC International (#001169) in accordance with the Guide for the Care and Use of Laboratory Animals, 8th edition, NRC (2010).

Note that full information on the approval of the study protocol must also be provided in the manuscript.

Human research participants

Policy information about studies involving human research participants

Population characteristics	Participant was health subject (Height: over 180 cm)
Recruitment	Human participant was recruited by online. There were no self-selection biases or other biases.
Ethics oversight	The study was approved by the Institutional Review Board of the Seoul National University Hospital, Seoul, Korea (#2104-230-1217).

Note that full information on the approval of the study protocol must also be provided in the manuscript.
